# National public health law: a role for WHO in capacity-building and promoting transparency

**DOI:** 10.2471/BLT.15.164749

**Published:** 2016-05-03

**Authors:** Géraldine Marks-Sultan, Feng-jen Tsai, Evan Anderson, Florian Kastler, Dominique Sprumont,, Scott Burris

**Affiliations:** a Institut de Droit de la Santé, Université de Neuchâtel, Avenue du 1er-Mars 26, 2000 Neuchâtel, Switzerland.; b Masters Program in Global Health and Development, Taipei Medical University, Taiwan, China.; c Center for Public Health Initiatives, University of Pennsylvania, Philadelphia, United States of America (USA).; d Health and Law Institute, University of Paris Descartes, Paris, France.; e Beasley School of Law, Temple University, Philadelphia, USA.

## Abstract

A robust health infrastructure in every country is the most effective long-term preparedness strategy for global health emergencies. This includes not only health systems and their human resources, but also countries’ legal infrastructure for health: the laws and policies that empower, obligate and sometimes limit government and private action. The law is also an important tool in health promotion and protection. Public health professionals play important roles in health law – from the development of policies, through their enforcement, to the scientific evaluation of the health impact of laws. Member States are already mandated to communicate their national health laws and regulations to the World Health Organization (WHO). In this paper we propose that WHO has the authority and credibility to support capacity-building in the area of health law within Member States, and to make national laws easier to access, understand, monitor and evaluate. We believe a strong case can be made to donors for the funding of a public health law centre or unit, that has adequate staffing, is robustly networked with its regional counterparts and is integrated into the main work of WHO. The mission of the unit or centre would be to define and integrate scientific and legal expertise in public health law, both technical and programmatic, across the work of WHO, and to conduct and facilitate global health policy surveillance.

## Résumé

Avoir une infrastructure sanitaire solide dans chaque pays est la meilleure stratégie à long terme de préparation aux urgences sanitaires mondiales. Il s'agit non seulement des systèmes de santé et de leurs ressources humaines, mais aussi de l'infrastructure juridique des pays en matière de santé: les lois et les politiques qui permettent, obligent et parfois limitent l'action du gouvernement et du secteur privé. La législation est également un outil important pour la promotion et la protection de la santé. Les professionnels de la santé publique jouent un rôle important vis-à-vis de la législation sanitaire - de l'élaboration des politiques à leur application, en passant par l'évaluation scientifique de l'impact des lois sur la santé. Les États membres sont déjà chargés de faire part de leurs lois et règlements nationaux en matière de santé à l'Organisation mondiale de la Santé (OMS). Le présent article suggère que l'OMS, qui dispose de l'autorité et de la crédibilité pour le faire, participe au renforcement des capacités dans le domaine de la législation sanitaire des États membres et facilite l'accès, la compréhension, le contrôle et l'évaluation des lois nationales. Des arguments convaincants peuvent être présentés aux bailleurs de fonds pour le financement d'un centre ou d'une unité de législation de la santé publique qui disposerait du personnel nécessaire, entretiendrait de solides relations avec ses homologues régionaux et serait intégré aux principaux travaux de l'OMS. La mission de cette unité ou de ce centre consisterait à définir et intégrer l'expertise scientifique et juridique dans la législation de la santé publique, sur le plan technique et programmatique, dans le cadre des travaux de l'OMS, et à réaliser et faciliter la surveillance des politiques sanitaires mondiales.

## Resumen

Una infraestructura sanitaria firme en todos los países es la estrategia de preparación más eficaz a largo plazo para tratar las emergencias sanitarias en todo el mundo. Esto no solo incluye los sistemas sanitarios y sus recursos humanos, sino también la infraestructura legal para la salud de cada país: las leyes y políticas que facultan, obligan y, en ocasiones, limitan la acción privada y del gobierno. La legislación también es una herramienta importante para el fomento y la protección de la salud. Los profesionales de la salud pública tienen funciones fundamentales en la legislación sanitaria: desde el desarrollo de políticas a través de su puesta en vigor, hasta la evaluación científica del impacto sanitario de las leyes. Ya se ha encomendado a los Estados Miembros que comuniquen sus leyes y normativas de salud nacionales a la Organización Mundial de la Salud (OMS). Este artículo propone que la OMS tenga la autoridad y credibilidad para dar apoyo a la creación de capacidad de las leyes sanitarias de los Estados Miembros, y para facilitar el acceso, la comprensión, la supervisión y la evaluación de las leyes nacionales. Creemos que hay argumentos firmes a favor de las contribuciones para la financiación de un centro o unidad de derecho de salud pública que cuente con el personal adecuado, tenga un sistema de redes sólido con sus contrapartes regionales y esté integrado con el trabajo principal de la OMS. La misión de la unidad o centro sería definir e integrar la experiencia científica y legal en la ley de salud pública, tanto en el aspecto técnico como programático, a través del trabajo de la OMS, así como dirigir y facilitar el control de las políticas sanitarias globales.

## ملخص

إن إنشاء بنية تحتية قوية للصحة في كل بلد يمثل استراتيجية التأهب الأكثر فعالية على المدى الطويل لحالات الطوارئ الصحية العالمية. وهذا الأمر لا يشمل فقط أنظمة الصحة والموارد البشرية الخاصة بها، وإنما يمتد أيضًا ليشمل البنية التحتية القانونية للصحة في البلدان، بما يشمل: القوانين والسياسات التي تعمل على تمكين وفرض الإجراءات الحكومية والخاصة وأحيانًا الحد من تأثيرها. ويمثل القانون أيضًا أداةً مهمة في مجال حماية الصحة وتشجيع الحفاظ عليها. ويؤدي المتخصصون في مجال الصحة العامة أدوارًا هامة في قانون الصحة – بداية ًمن وضع السياسات، حتى إنفاذها، ونهايةً بإجراء التقييم العلمي عن التأثير الصحي للقوانين. تم إلزام الدول الأعضاء بالفعل بإعلام منظمة الصحة العالمية بقوانينها الصحية الوطنية ولوائحها التنظيمية. نقترح في هذه الدراسة منح منظمة الصحة العالمية الصلاحية والمصداقية لدعم بناء القدرات في مجال القوانين الصحية داخل نطاق الدول الأعضاء، وتسهيل الاطلاع على القوانين الوطنية وفهمها ومتابعتها وتقييمها بشكلٍ أكبر. ونحن نعتقد أنه يمكن تقديم عرض مُقنِع للجهات المانحة لتمويل مركز أو وحدة قانون الصحة العامة، التي يتواجد فيها عدد كافٍ من الموظفين، والتي يتم توصيلها بشبكة قوية بنظيراتها الإقليمية، مع دمجها في نطاق العمل الرئيسي لمنظمة الصحة العالمية. وعلى أن تتمثل مهمة الوحدة أو المركز في تحديد ودمج الخبرات العلمية والقانونية في قانون الصحة العامة، لكل من الخبرات التقنية والبرمجية، وذلك عبر نطاق الأعمال التي تؤديها منظمة الصحة العالمية، والعمل على إجراء أعمال مراقبة السياسة الصحية العالمية وتسهيلها.

## 摘要

在每个国家建立稳健的卫生基础设施是应对全球性卫生突发事件最有效的长期准备战略。 这不仅包括卫生系统及其人力资源,而且还包括各国有关卫生的法律基础设施: 赋予权力、义务并且有时限制政府和民间活动的法律和政策。 法律也是促进卫生和卫生防护的重要工具。 从政策的制定、执行,到科学评估法律对卫生的影响——公共卫生专业人员在卫生法律中发挥重要的作用。 成员国必须将其国家卫生法律和法规传达给世界卫生组织。 在本文中,我们建议世界卫生组织凭借其权威性和公信力在成员国中支持卫生法律领域的能力建设,并且使国家法律更加便于查阅、了解、监控和评估。 我们认为应强力主张捐助方为公共卫生法律中心或部门提供经费,使这些中心或部门配备足够的工作人员,与区域内的同行稳健地联网并且融入世界卫生组织的主要工作中。 这些部门或中心的任务将是在世界卫生组织的整个工作范围内确定与整合公共卫生法律中的科学与法律专业知识(包括技术和程序知识),并且开展和促进全球卫生政策监督。

## Резюме

Поддержание сильной инфраструктуры системы здравоохранения в каждой стране является наиболее эффективной долгосрочной стратегией обеспечения готовности к чрезвычайным ситуациям в области мирового здравоохранения. В данном случае под инфраструктурой понимаются не только системы здравоохранения и их людские ресурсы, но и правовая инфраструктура стран в области здравоохранения, а именно законы и положения, которые расширяют возможности, налагают обязательства и иногда вводят ограничения для деятельности правительств и частных лиц. Законодательство также является важным инструментом укрепления и охраны здоровья. Профессиональные работники сферы общественного здравоохранения играют важные роли в сфере здравоохранительного права, от разработки стратегий, их реализации до научной оценки воздействия законов на здоровье. Государства-участники уже приняли на себя обязательство сообщать Всемирной организации здравоохранения (ВОЗ) о своих национальных законах и правилах, относящихся к области здравоохранения. В данной статье высказывается предположение, что ВОЗ обладает достаточным авторитетом и репутацией для поддержки наращивания потенциала в сфере здравоохранительного права государств-участников и для облегчения доступа к внутренним законам, их понимания, отслеживания и оценки. Авторы уверены, что существуют веские доводы, которые можно привести донорам, в пользу финансирования ориентированного на сферу общественного здравоохранения юридического центра или группы, которые располагали бы кадровым составом соответствующего уровня подготовки, прочными связями со своими региональными партнерами и были бы вовлечены в основную деятельность ВОЗ. Миссия этой группы или центра заключалась бы в определении научных и юридических знаний (как технических, так и программных), их внедрении в законодательство, относящееся к общественному здравоохранению, во всех областях деятельности ВОЗ, а также в осуществлении надзора за политикой в сфере мирового здравоохранения и содействии ему.

## Introduction

At times of global health emergencies, such as the Ebola and Zika virus disease outbreaks, the world looks to the World Health Organization (WHO) for leadership. When problems are perceived in the handling of a crisis,^1^^,^^2^ there are sometimes calls for changes to the international legal instruments that set the agency’s powers, duties and procedures for action, particularly the International Health Regulations (IHR).^3^^,^^4^ The IHR, which are binding on all WHO Member States, are a logical target for legal concern, since they set out basic rules of international law requiring countries to strengthen their national surveillance and response capacities, and to share important information with the global community.^5^ We believe, however, that a focus on international law is mistaken. We argue in this paper that the most critical legal need for action in global public health generally, including emergency preparedness, is at the national level. Many nations lack the basic laws and regulations needed to comply with IHR obligations and to support effective public health systems, or have laws that are outdated or poorly designed. Moreover, action to improve legal infrastructure is hindered by the inaccessibility of information about the health laws of nations, which reduces transparency and nations’ accountability for meeting their international obligations. WHO is in a position to provide leadership in improving national legal compliance, transparency and accountability, and in this paper we make the case that it is both legally appropriate and feasible for WHO to do so.

## National legal infrastructure

As WHO and others have stressed, promoting a robust health infrastructure in every country is the most effective long-term preparedness strategy for global health emergencies.^1^^,^^6^ Health infrastructure includes not only the physical structures of public health agencies, clinics and hospitals and the human resources to operate them, but also countries’ legal infrastructure – the laws and policies that empower, obligate and limit government and private action concerning health. A health emergency tests how effectively regulatory strategies, social contract principles and human rights norms have been embodied in the written laws of a country, and how closely, in turn, those legal embodiments guide action.^7^^,^^8^ Disease outbreaks, for example, require a wide range of actions (e.g. disease reporting, surveillance, quarantine, social distancing, curfews, import of medical supplies and personnel, and vector control), all of which are effected through, or subject to, national laws. Governments are also obliged to protect the human rights of individuals affected by an outbreak. This requirement in the IHR for preparedness can be understood as a requirement on Member States to develop the laws and regulations needed to carry out these measures.^9^ Direct assistance to countries to help them develop their own legal infrastructure and capacity would be more helpful to emergency response than any changes in the wording of the IHR, which were already substantially revised in 2005.

Measures to handle emergency response in a health crisis are only one facet of public health law. The law is also an important tool in health promotion and protection.^10^ Laws are central, for example, to a country’s strategy to improve road safety,^11^ reduce tobacco use and manage lifestyle-related chronic diseases.^12^^,^^13^ Virtually all the major public health achievements of the last century, from universal immunization to tobacco control, depended on legal interventions.^14^ An initiative called Health in All Policies, which aims to identify and address health issues across all government sectors and policy topics, exemplifies the consensus that laws matter to health even when they target non-health issues.^15^

Public health law, broadly defined, includes laws that are intended as health interventions, laws that define the powers, duties and boundaries of health agencies and systems, and laws that have an impact on health but were not enacted with population health in mind.^16^ As the role of public health practitioners and researchers in the law has grown, a transdisciplinary model of public health law has emerged that integrates legal and scientific expertise in public health law research and practice.^17^ The law is such an integral part of public health that public health professionals, and not only lawyers, are working with the law on a regular basis, playing important roles in the development, enforcement and evaluation of health-related laws. Public health professionals and the communities they serve have much to gain from training and support for these law-related activities.

Across the work of WHO, the law of an individual Member State may act as an intervention tool,^18^ as an important influence on the health of the population^19^ or as a barrier to action.^20^^,^^21^ While the existence of specific laws is not sufficient in itself for a strong health system generally or for effective legal interventions in specific threats, it is clear that the law matters for effective health practice. Yet currently the national legal situation in most countries at risk of health crisis is opaque.^22^ Although in most countries the texts of laws are theoretically available, identifying specific laws can be difficult in practice. More importantly, in the midst of a health crisis, decision-makers need an immediate understanding of the legal situation without waiting for lawyers to search for the relevant legal texts. Furthermore, having the legal information of a country available in an unstructured format can be a hindrance to the task of comparing laws across countries, identifying country-level strengths and deficiencies, or monitoring changes over time.^23^

## Stronger role for WHO

We propose that WHO has the authority and credibility to support capacity-building in the area of health law within Member States, and to make national laws and regulations easier for all interested stakeholders to access, understand, monitor and evaluate. WHO’s constitutional mandate is the “attainment by all peoples of the highest possible level of health”^24^ and it was given open-ended authority “to take all necessary actions” to advance this objective. Specific articles of IHR 2005 reinforce this by stating that WHO will collaborate with Member States in the provision or facilitation of technical cooperation and logistic support (Article 44), and will assist, on request, to develop, strengthen and maintain their core public health capacities (Article 5).^5^ Although not expressly mentioned within the text of the IHR, national legislation, policy and financing is defined by WHO as a key indicator of progress in the development of IHR core capacities by countries.^25^

The law is a normal mode of public health intervention, and so working with the law falls within WHO’s mission.^22^^,^^26^ Although WHO has never had a distinct public health law programme or internal law centre, it has supported legal collaborating centres and done vital work in matters as diverse as access to opioids for pain care and road safety.^11^^,^^27^ Such work is essential in assisting countries to develop and implement healthy public policies – and is an appropriate exercise of WHO’s authority. The WHO Constitution specifically grants it the legal authority to propose conventions, agreements and regulations. These explicit legal powers exist alongside the general authority to engage in assistance activities to improve health services and support informed public opinion on health matters in Member States. It is thus consistent with both the WHO Constitution and prevailing best practice in global public health law for WHO to provide the same kind of technical expertise and standard guidance on the law that it provides with respect to other tools and strategies of health promotion and protection for Member States.

WHO’s legal mandate extends to addressing a lack of transparency in national public health laws. Article 63 of the Constitution obliges Member States to communicate to WHO their important laws, regulations, official reports and statistics pertaining to health. This obligation clearly reflects the importance of national laws to WHO’s mission, and should be interpreted to mean more than countries simply sending copies of their legislative documents to be filed at WHO headquarters. Transparency of national laws, and accessibility to national legal information, is crucial to effective international coordination in health and to the supportive role of WHO in providing needed expertise and guidance. The authority to collect these legal texts places WHO in a unique position to support better access to Member States’ national health laws for the purposes of research, emergency response, public information and technical assistance. The challenge for WHO has always been to get compliance with this mandate, and then to manage the resulting flow of legal texts. WHO published the *International Digest of Health Legislation* in paper form for many years and then on the Internet,^28^ although the website appears to have been offline for some time.^29^ Advances in Internet technology and in public health law research methods now offer a practical solution to the problem of managing the information within WHO’s reach.^30^

Progress must begin with a conversation about the goals and uses of health law information. A database of all the health legislation of Member States would in theory meet the need for national legal transparency and access to legal information. There are, however, problems with this sort of comprehensive database.^23^ First, a large amount of the data collected will not be relevant or applicable to any current, important public health problem. Second, gathering information together in one place does not automatically facilitate the extraction of relevant knowledge. Finally, the emergence of the Internet and a general trend towards digitizing legal information means that the legal information world of 2016 is quite different from that of 1946, when WHO adopted its Constitution, or even 1986, when the *International Digest of Health Legislation* was still being printed.^31^ More and more national laws are being posted on the Internet, so that combining these in one database, though helpful, is a less needed service than it once was, and may become even less relevant in the future.

Finally, the case for WHO taking a role in collecting legal information and supporting Member States’ legal capacity is bolstered by the fact that other agencies and organizations in the United Nations system already do so and have operational legal databases. The Food and Agriculture Organization has a database on national legislations and policies relating to food, agriculture and renewable natural resources,^32^ the International Labour Organization has a database of national labour, social security and related human rights legal texts^33^ and the Global Database on Occupational Safety and Health Legislation that reports on occupational safety and health national laws, regulations and policies,^34^ the World Intellectual Property Organization provides texts for copyrights, trademarks and patent legislations,^35^ and the United Nations Office on Drugs and Crime has an online legal library of drug legislation to assist in monitoring the implementation of international conventions in the field.^36^

## Towards a plan of action

In law, as in other areas of its work, WHO shares the field with organizations that have their own expertise and resources. For example, the International Development Law Organization has been working with WHO and others to build capacity in and promote greater understanding of public health law.^37^ The Global Health Security Agenda is working to strengthen legal emergency preparedness at the national level.^38^^,^^39^ A commission on global health and the law, sponsored by the O’Neill Institute for National and Global Health Law at Georgetown University, Washington, United States of America, a WHO collaborating centre on public health law and human rights, is writing recommendations for strengthening the field.^39^^,^^40^ So what is the most useful role for WHO in public health law? We suggest focusing on two gaps that WHO is in the best position to fill: (i) the need for more support to countries in assessing and improving their health law infrastructure; and (ii) the need for leadership to improve global access to national law for research, emergency response, public information and technical assistance purposes through policy surveillance.

### Legal capacity-building

Helping countries to strengthen their legal infrastructure entails both understanding the law and how it works and knowing what laws are empirically associated with better health outcomes. In areas such as tobacco control, the work of WHO exhibits a high degree of integration of legal and scientific expertise. Legal experts at WHO headquarters are also doing excellent work in many other areas including noncommunicable disease prevention, legal aspects of health systems and road safety. In addition to WHO’s collaboration with the International Development Law Organization,^37^ there are both new and well-established nodes of legal expertise in WHO’s regional offices. Overall, however, WHO needs to invest more in the legal capacity of its headquarters and in the regions, not just by employing more lawyers but in capacity-building for health professionals without legal training who are working in policy development, monitoring and evaluation. Given the challenges of finding the necessary financial resources for this, we believe a strong case can be made to donors for the funding of a public health law centre or unit, that has adequate staffing, is robustly networked with its regional counterparts and is integrated into the main work of WHO.

The mission of the unit or centre would be to define and integrate scientific and legal expertise in public health law, both technical and programmatic, across the work of WHO. The Public Health Law Program of the United States Centers for Disease Control and Prevention (CDC) is a good model. It provides legal research and analysis services within CDC and to local, state and tribal health agencies. It also supports international legal support projects.^41^ The proposed unit at WHO could provide public health legal services to other parts of the agency, conduct monitoring and evaluation projects, offer internal and external training, and develop tools and methods for both legal services and law-related scientific and health practice functions. Like the Public Health Law Program, a WHO law programme could also work with and support an informal network of external health law programmes that are engaged in building the field of public health law and conducting projects relevant to WHO’s work.

### Policy surveillance

Facilitating transparency for countries’ health laws requires more than collecting and providing access to a mass of legal text. Thorough research and analysis are needed to turn the text into concise, actionable legal knowledge that is relevant to decision-makers and is comparable across countries and over time. In recent years, policy surveillance has emerged as an approach to manage large quantities of data. Policy surveillance in this context is the “ongoing, systematic collection, analysis, interpretation and dissemination of information about a given body of public health law and policy”.^42^ The policy surveillance approach differs from a simple database of legal texts in several ways. The approach is selective, capturing only provisions that are expected to be of ongoing significance, for which up-to-date information is required for planning, capacity-building, tracking progress or conducting evaluations of legal impact.^43^ It also uses scientific methods of collecting and coding legal information, including the use of specified quality control processes within an explicit research protocol, so that the results are highly credible, but also of a quality and in a form that can be used in evaluation research.^44^ Modern database software, including at least one program developed for multi-jurisdictional legal research, and the Internet allow this kind of digitized, structured legal data to be published on interactive websites that allow users to not only access the data but also create custom reports comparing the important features of the law across countries or tracking trends over time.^30^ Examples of such surveillance sites include the Alcohol Policy Information System (a project of the United States National Institute on Alcohol Abuse and Alcoholism),^45^ the Prescription Drug Abuse Policy System (funded by the United States National Institute on Drug Abuse),^46^ the Law Atlas (a programme of the Public Health Law Research Program, funded by the Robert Wood Johnson Foundation)^47^ and the WORLD Policy Analysis Center (based at the Fielding School of Public Health of the University of California Los Angeles).^48^

WHO’s leadership role in global health, and its official access to Member States’ health laws, puts it in a position to take a lead in establishing policy surveillance of the national laws most important to health. WHO is already participating in road safety^49^ and tobacco control^50^ projects that entail the ongoing surveillance of national public health laws in its Member States. Potential partners and collaborators include the many groups collecting and analysing national health legislation, such as the current effort under the Global Health Security Agenda.^39^ WHO can use its convening power and its broad topical expertise to bring together a group of experts and funders to consider the best model for global health policy surveillance. The options are many, including a single WHO-based portal, a collaboration along the lines of Cochrane or an informal network of providers using shared methods and data structures to maximize the quality, utility and exchange of legal data.^23^^,^^30^

## Conclusion

Nations have made strong commitments to action for health in instruments such as the Constitution of the WHO, the IHR and the Framework Convention on Tobacco Control. To fulfil these commitments, countries must enact national legislation to carry them out. Once enacted, health law can be a powerful tool for changing unhealthy behaviour and environments and for coordinating the work of health systems. A modern, effective public health agency must have the legal expertise and the ability across its staff to support policy development, capacity-building and scientific impact evaluation. It must know what is authorized by national law and be in a position to evaluate and identify needed reforms. In this paper, we have suggested that WHO develop a programme for public health law capacity-building and policy surveillance to ensure continuous and organized efforts to help Member States to strengthen their legal infrastructure. Modern public health cannot be effective without laws, and WHO cannot achieve its goals unless it can help all Member States to develop and implement the legal and regulatory tools they need.

## References

[1] Ebola: what lessons for the International Health Regulations? Lancet. 2014 10 11;384(9951):1321. 10.1016/S0140-6736(14)61697-4 25306562 PMC7136992

[2] Philips M, Markham Á. Ebola: a failure of international collective action. Lancet. 2014 9 27;384(9949):1181 10.1016/S0140-6736(14)61606-8 25218774

[3] WHO leadership statement on the Ebola response and WHO reforms [Internet]. Geneva: World Health Organization; 2015. Available from: http://www.who.int/csr/disease/ebola/joint-statement-ebola/en/ [cited 2014 Apr 29].

[4] Ebola virus disease outbreak and follow-up to the Special Session of the Executive Board on Ebola. Outcome of the drafting group [Internet]. Geneva: World Health Organization; 2015 (A/68/CONF./5). Available from: http://apps.who.int/gb/ebwha/pdf_files/WHA68/A68_ACONF5-en.pdf [cited 2014 Apr 29].

[5] International Health Regulations. 2005. 2nd ed. Geneva: World Health Organization; 2008. Available from: http://apps.who.int/iris/bitstream/10665/43883/1/9789241580410_eng.pdf [cited 2014 Apr 14].

[6] Gostin LO. Ebola: towards an international health systems fund. Lancet. 2014 10 11;384(9951):e49–51. 10.1016/S0140-6736(14)61345-3 25201591

[7] Sapsin JW, Gostin LO, Vernick JS, Burris S, Teret SP. SARS and international legal preparedness. Temple Law Rev. 2004;77(2):155–74.

[8] Rutkow L, Vernick JS, Thompson CB, Pirrallo RG, Barnett DJ. Emergency preparedness law and willingness to respond in the EMS workforce. Prehosp Disaster Med. 2014 8;29(4):358–63. 10.1017/S1049023X14000788 25046354

[9] Gostin L. Global health law. Cambridge: Harvard University Press; 2014 10.4159/9780674369870

[10] Burris S, Anderson E. Legal regulation of health-related behavior: a half century of public health law research. Annu Rev Law Soc Sci. 2013;9(1):95–117. 10.1146/annurev-lawsocsci-102612-134011

[11] Global status report on road safety 2015. Geneva: World Health Organization; 2015.

[12] Mensah GA, Goodman RA, Zaza S, Moulton AD, Kocher PL, Dietz WH, et al. Law as a tool for preventing chronic diseases: expanding the range of effective public health strategies. Prev Chronic Dis. 2004 1;1(1):A13. 15634375 PMC544536

[13] Mensah GA, Goodman RA, Zaza S, Moulton AD, Kocher PL, Dietz WH, et al. Law as a tool for preventing chronic diseases: expanding the spectrum of effective public health strategies. Prev Chronic Dis. 2004 4;1(2):A11. 15663886 PMC1183502

[14] Ten great public health achievements – United States, 1900–1999. MMWR Morb Mortal Wkly Rep. 1999 4 2;48(12):241–3. 10220250

[15] Baum F, Lawless A, Delany T, Macdougall C, Williams C, Broderick D, et al. Evaluation of Health in All Policies: concept, theory and application. Health Promot Int. 2014 6;29 Suppl 1:i130–42. 10.1093/heapro/dau032 25217350

[16] Burris S, Wagenaar AC, Swanson J, Ibrahim JK, Wood J, Mello MM. Making the case for laws that improve health: a framework for public health law research. Milbank Q. 2010 6;88(2):169–210. 10.1111/j.1468-0009.2010.00595.x 20579282 PMC2980343

[17] Burris S, Ashe M, Levin D, Penn M, Larkin M. A transdisciplinary approach to public health law: the emerging practice of legal epidemiology. Annu Rev Public Health. 2015 Nov 30. 11 30 26667606 10.1146/annurev-publhealth-032315-021841 PMC5703193

[18] Sebego M, Naumann RB, Rudd RA, Voetsch K, Dellinger AM, Ndlovu C. The impact of alcohol and road traffic policies on crash rates in Botswana, 2004-2011: a time-series analysis. Accid Anal Prev. 2014 9;70:33–9. 10.1016/j.aap.2014.02.017 24686164 PMC4697825

[19] Komro KA, Burris S, Wagenaar AC. Social determinants of child health: concepts and measures for future research. Health Behav Policy Rev. 2014;1(6):432–45. 10.14485/HBPR.1.6.1

[20] Yeager VA, Hurst D, Menachemi N. State barriers to appropriating public health emergency response funds during the 2009 H1N1 response. Am J Public Health. 2015 4;105(S2) Suppl 2:S274–9. 10.2105/AJPH.2014.302378 25689213 PMC4355722

[21] Mello MM, Powlowski M, Nañagas JMP, Bossert T. The role of law in public health: the case of family planning in the Philippines. Soc Sci Med. 2006 7;63(2):384–96. 10.1016/j.socscimed.2006.01.010 16488063

[22] Attaran A, Pang T, Whitworth J, Oxman A, McKee M. Healthy by law: the missed opportunity to use laws for public health. Lancet. 2012 1 21;379(9812):283–5. 10.1016/S0140-6736(11)60069-X 21982516

[23] Sprumont D. Options to streamline the reporting of and communication with Member States. Internal report to WHO on EB132/5 Add. 4. Neuchâtel: Institute of Health Law; 2013.

[24] Constitution of the World Health Organization [Internet]. Geneva: World Health Organization; 1946. Available from: http://www.who.int/governance/eb/who_constitution_en.pdf [cited 2014 Apr 14].

[25] IHR core capacity monitoring framework: checklist and indicators for monitoring progress in the development of IHR core capacities in States Parties. Geneva: World Health Organization; 2013.

[26] Gostin LO, Taylor AL. Global health law: a definition and grand challenges. Public Health Ethics. 2008 4 1;1(1):53–63.

[27] De Lima L, Bruera E, Joranson DE, Vanegas G, Cepeda S, Quesada L, et al. Opioid availability in Latin America: the Santo Domingo report progress since the declaration of Florianopolis. J Pain Symptom Manage. 1997 4;13(4):213–9. 10.1016/S0885-3924(96)00325-9 9136232

[28] International Digest of Health Legislation [Internet]. Geneva: World Health Organization; 2014. Available from: http://www.who.int/idhl-rils/index.cfm [cited 2014 Apr 26].

[29] Attaran A, Capron AM. Universal health coverage and health laws. Lancet. 2014 1 4;383(9911):25. 10.1016/S0140-6736(13)62724-5 24388300

[30] Burris S, Hitchcock L, Ibrahim JK, Penn M, Ramanathan T. Policy surveillance: a vital public health practice comes of age. J Health Polit Policy Law. 2016. Forthcoming. 10.1111/jlme.12210 27531941

[31] Burris S. Public health law monitoring and evaluation in a big data future. I/S J Law Policy Inf Soc. 2015;11(1):115–26. 10.1089/bsp.2014.3314 24641445

[32] FAOLEX legal office [Internet]. Rome: Food and Agriculture Organization; 2014. Available from: http://faolex.fao.org/faolex/index.htm [cited 2014 Apr 14].

[33] NATLEX database of national labour, social security and related human rights legislation [Internet]. Geneva: International Labour Organization; 2014. Available from: http://www.ilo.ch/dyn/natlex/natlex4.home [cited 2014 Apr 27].

[34] LEGOSH occupational safety and health global database [Internet]. Geneva: International Labour Organization; 2014. Available from: http://www.ilo.org/dyn/legosh/en/f?p=14100:1000:0:NO [cited 2014 Apr 14].

[35] WIPO Lex [Internet]. Geneva: World Intellectual Property Organization; 2014. Available from: http://www.wipo.int/wipolex/en/ [cited 2014 Apr 14].

[36] Legal library on drug control [Internet]. Vienna: United Nations Office on Drugs and Crime; 2014. Available from: https://www.unodc.org/enl/ [cited 2014 Apr 14].

[37] Legal tools for healthy diets and physical activity [Internet]. Rome: International Development Law Organization; 2015. Available from: http://www.idlo.int/what-we-do/initiatives/legal-tools-healthy-diets-and-physical-activity [cited 2014 Apr 14].

[38] Inglesby T, Fischer JE. Moving ahead on the global health security agenda. Biosecur Bioterror. 2014 Mar-Apr;12(2):63–5. 10.1089/bsp.2014.3314 24641445

[39] Global health – CDC and the Global Health Security Agenda [Internet]. Atlanta: Centers for Disease Control and Prevention. Available from: http://www.cdc.gov/globalhealth/security/ [cited 2014 Apr 14].

[40] Gostin LO, Monahan JT, DeBartolo MC, Horton R. Law’s power to safeguard global health: a Lancet-O’Neill Institute, Georgetown University Commission on Global Health and the Law. Lancet. 2015 4 25;385(9978):1603–4. 10.1016/S0140-6736(15)60756-5 25911170

[41] Public Health Law Program [Internet]. Atlanta: Centers for Disease Control and Prevention; 2014. Available from: http://www.cdc.gov/phlp/index.html [cited 2014 Apr 14].

[42] Chriqui JF, O’Connor JC, Chaloupka FJ. What gets measured, gets changed: evaluating law and policy for maximum impact. J Law Med Ethics. 2011 3;39 Suppl 1:21–6. 10.1111/j.1748-720X.2011.00559.x 21309890

[43] Presley D, Reinstein T, Webb-Barr D, Burris S. Creating legal data for public health monitoring and evaluation: delphi standards for policy surveillance. J Law Med Ethics. 2015 Spring;43(s1) Suppl 1:27–31. 10.1111/jlme.12210 25846159

[44] Anderson E, Tremper C, Thomas S, Wagenaar AC. Measuring statutory law and regulations for empirical research. In: Wagenaar A, Burris S, editors. Public health law research: theory and methods. San Francisco: John Wiley & Sons; 2013 pp. 237–60.

[45] Alcohol policy information system [Internet]. Bethesda: National Institute on Alcohol Abuse and Alcoholism; 2014. Available from: http://alcoholpolicy.niaaa.nih.gov/ [cited 2014 Apr 14].

[46] Prescription Drug Abuse Policy System [Internet]. Philadelphia: Legal Science LLC; 2014. Available from: http://www.pdaps.org [cited 2014 Apr 26].

[47] LawAtlas: the policy surveillance portal [Internet]. Princeton: Robert Wood Johnson Foundation; 2014. Available from: http://lawatlas.org [cited 2014 Apr 14].

[48] WORLD Policy Analysis Center [Internet]. Los Angeles: Fielding School of Public Health, University of California Los Angeles; 2014. Available from: http://worldpolicycenter.org/ [cited 2014 Apr 14].

[49] Toroyan T. Global status report on road safety 2015. Geneva: World Health Organization; 2015.

[50] Framework Convention on Tobacco Control implementation database [Internet]. Geneva: World Health Organization; 2016. Available from: http://apps.who.int/fctc/implementation/database/ [cited 2014 Apr 26].

